# Supplementary material for: Inferring Potential Cancer Driving Synonymous Variants
Source: Genes (Basel). 2022 Apr 27;13(5):778. doi: 10.3390/genes13050778 (PMC9140830; doi:10.3390/genes13050778)
Supplement: Supplementary file 1 [file genes-13-00778-s001.zip › Supplementary_Materials/Supplementary_Figures.pdf]

Supplementary Figures

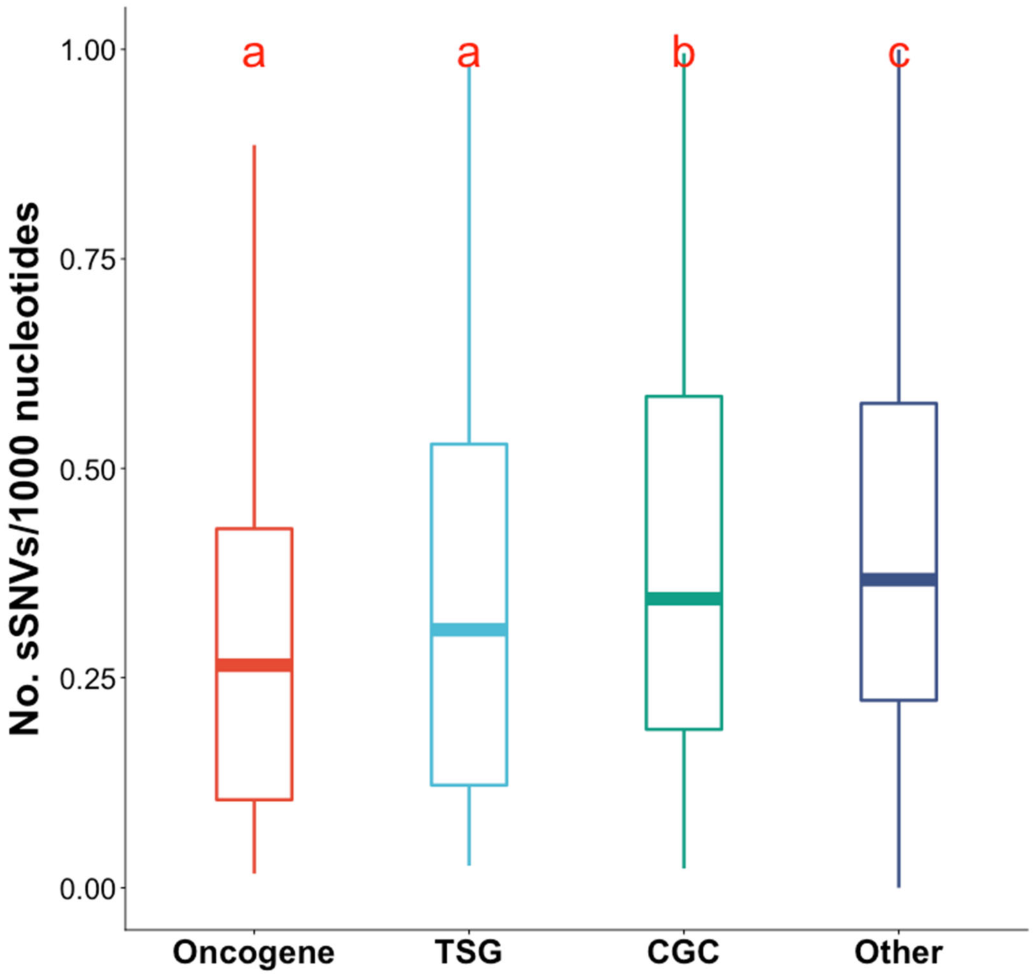

Supplementary Figure S1. Gene sSNV burden by gene type. Gene sSNV burden is the number of sSNVs normalized by length of gene coding region. The box plots indicate average sSNV burden of genes from the COSMIC database. Gene types are: oncogene, TSG (tumor suppressor gene), CGC (Cancer Gene Census), and other (none of the above). Kruskal-Wallis test (p-value<2.2e-16) was conducted and the null hypothesis that all groups follow the same distribution was rejected. A post-hoc Dunn test for pairwise comparisons was also performed, indicating that Oncogene and TSG gene burden distributions were similar, but not CGC or Other gene distributions (in figure: groups having different letter notation are statistically different).

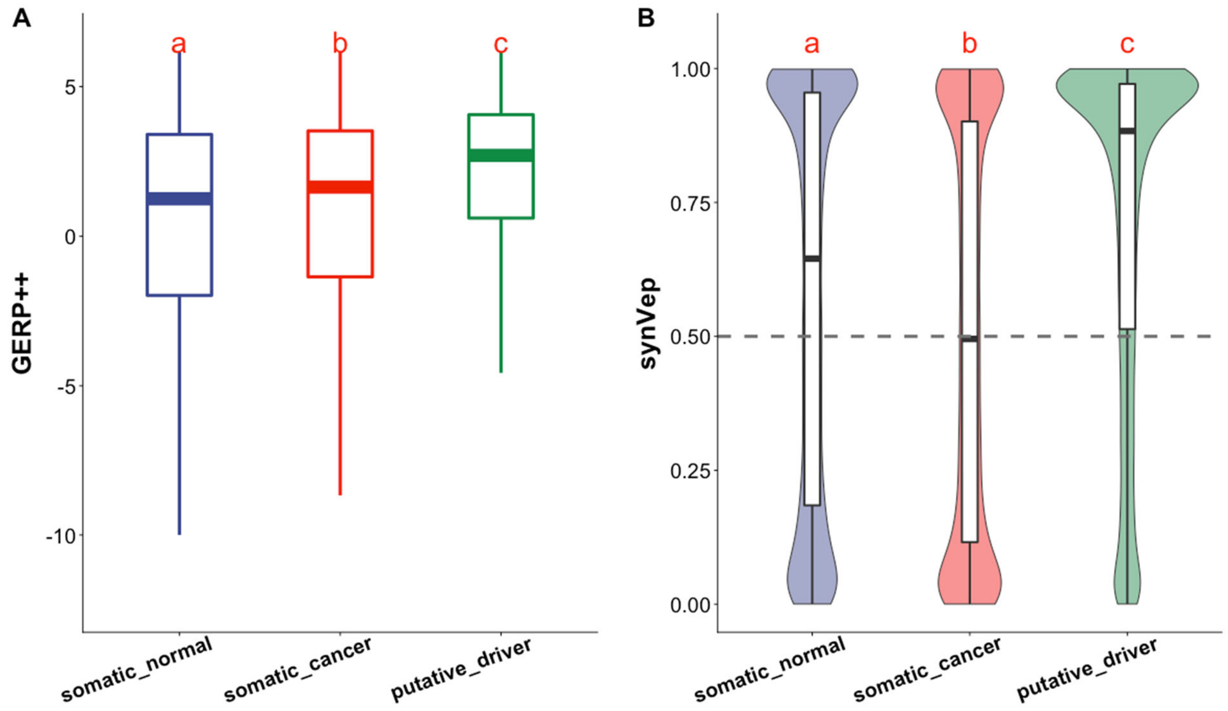

Supplementary Figure S2. Variation in conservation and synVep predictions of the *somatic normal*, *somatic cancer*, and *putative driver* sSNVs. In **(A)** GERP++ scores are used to measure conservation level. In **(B)**, for synVep predictions a score >0.5 (gray dashed line) means the variant is predicted to have an effect. For each panel, Kruskal-Wallis test (p-value<2.2e-16) was conducted (null hypothesis that all groups follow the same distribution is rejected), followed by post-hoc Dunn test for pairwise comparisons: groups having different letter notation are statistically different.

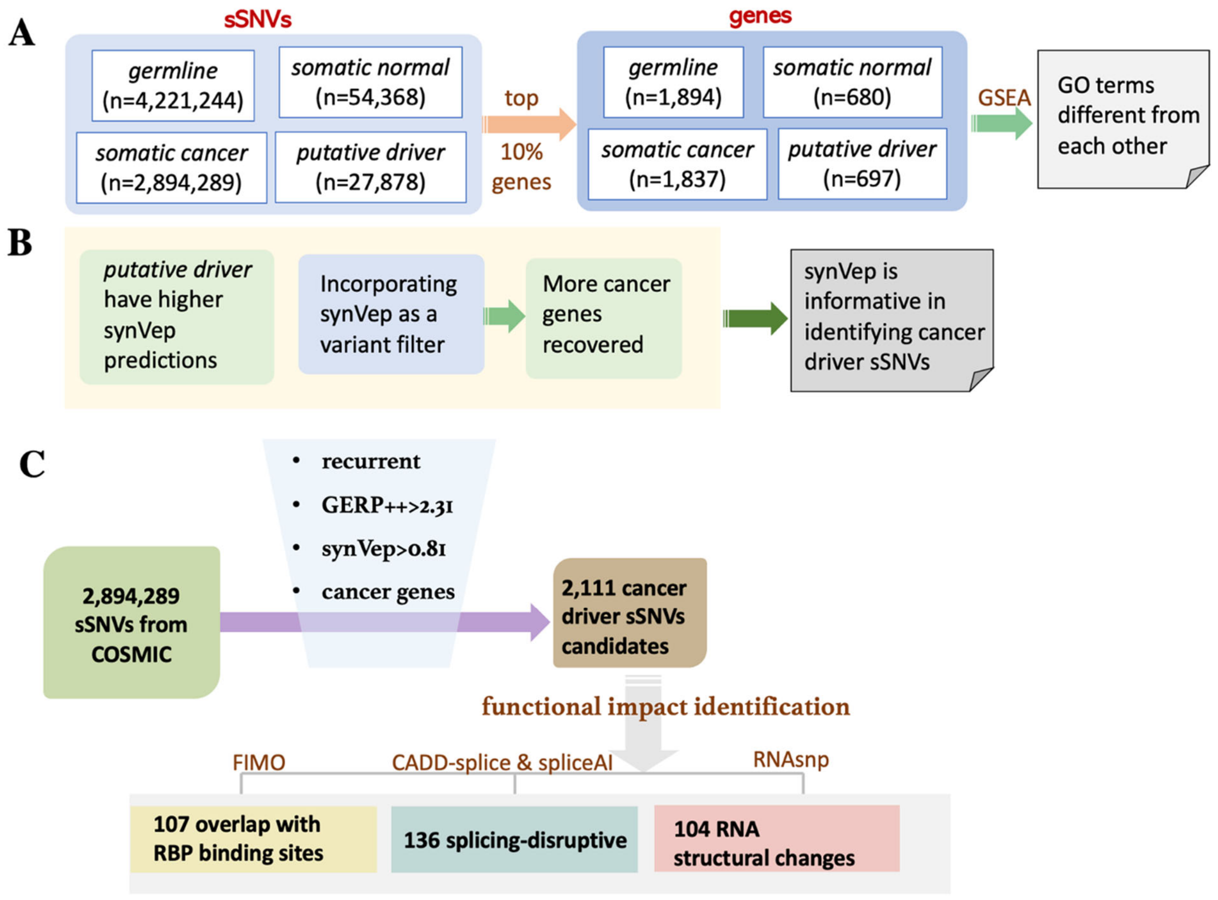

Supplementary Figure S3. Overview of the analysis and procedures of identifying potential cancer driving sSNVs. In panel A, we first showed the four categories of sSNVs () are fundamentally different with respect to underlying different gene ontologies. In panel B, we then evaluated the synVep distributions on the four categories of sSNVs, as well as the contribution of synVep to recover cancer genes, demonstrating the utility of synVep in identifying cancer driving sSNVs. In panel C, we applied multiple filters, including synVep, to identify potential cancer driving sSNVs; and finally, we identified the responsible mechanisms for these cancer driver candidates.

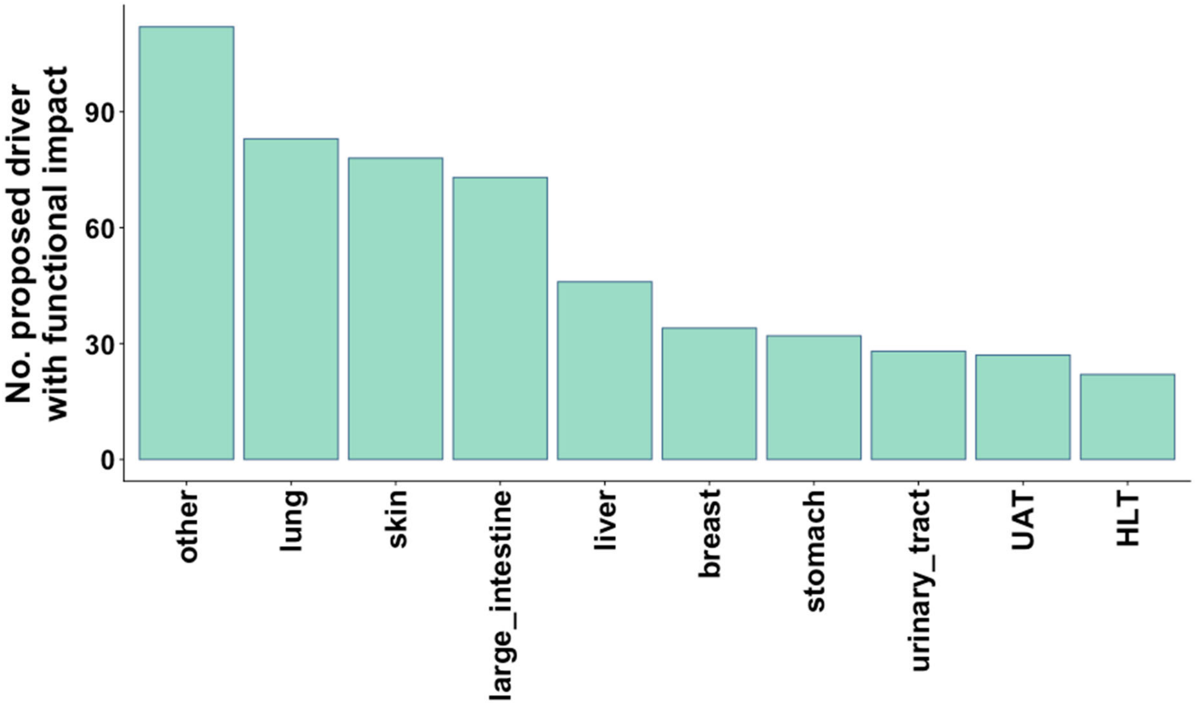

Supplementary Figure S4. Distribution of *proposed driver* sSNVs with identified functional impacts by cancer primary site. The “other” group aggregates all other cancer primary sites with less than 4 *driver* sSNVs with identified functional impacts.
